# Ground-Based Remote Standoff Laser Spectroscopies and Reflectance Spectral Imaging for Multimodal Analysis of Wall Painting Stratigraphy

**DOI:** 10.1021/acs.analchem.4c05264

**Published:** 2024-11-13

**Authors:** Yu Li, Amelia Suzuki, C. S. Cheung, Sotiria Kogou, Haida Liang

**Affiliations:** Imaging and Sensing for Archaeology, Art History and Conservation (ISAAC) Lab, School of Science and Technology, Nottingham Trent University, Nottingham NG11 8NS, U.K.

## Abstract

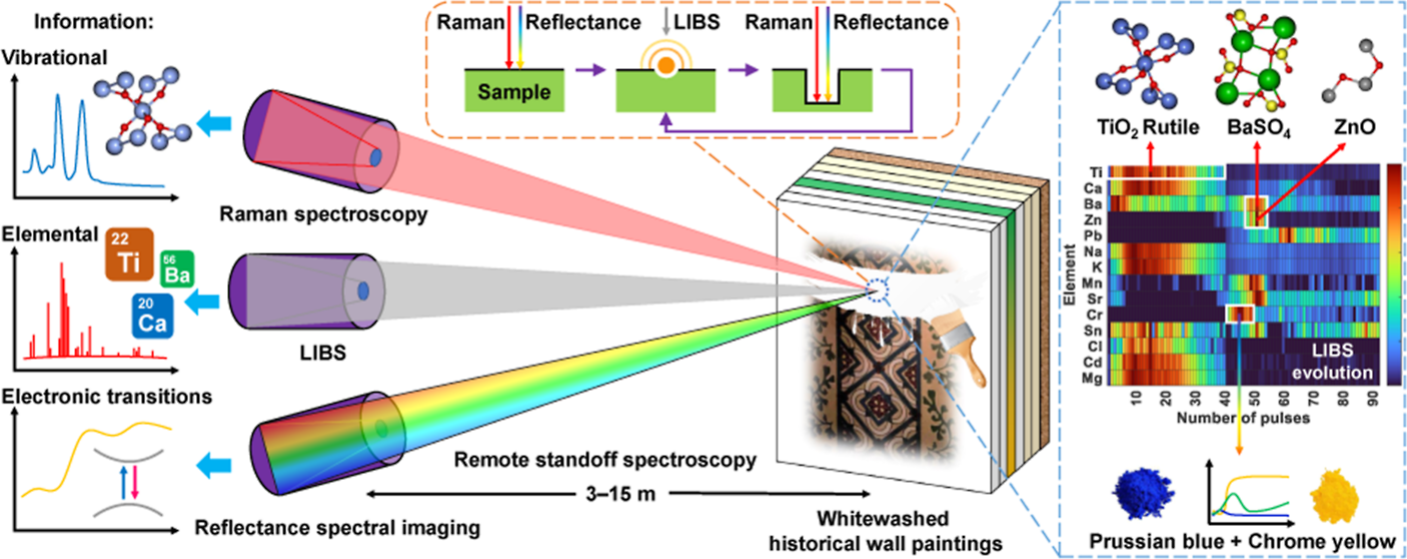

This paper presents a novel multimodal remote sensing setup to analyze the complex stratigraphy of historical wall paintings at distances of order 10 m. The proposed method enables comprehensive investigation of the chemical composition of multilayer paint stratigraphy by combining standoff laser-induced breakdown spectroscopy for elemental profiling with noninvasive standoff Raman spectroscopy and visible and near-infrared (400–900 nm) reflectance spectral imaging for depth-resolved complementary material characterization from a range of distances with instruments and operators located on stable ground. Following proof-of-concept laboratory tests, the feasibility and effectiveness of this standoff analytical approach is demonstrated through field analysis of a whitewashed historical wall painting, successfully identifying at least seven distinct layers from a distance of 7 m. The remote sensing method presented here can also be applied to other scientific and industrial domains to characterize the chemical composition of layered materials at a distance.

## Introduction

Historical wall paintings are heterogeneous multilayer systems that contain information about history, artistic techniques, original and degraded materials, and past conservation treatments. Their characterization is particularly challenging due to the lack of a priori knowledge regarding the original materials and the degradation history, compounded by their often inaccessible locations, such as high ceilings and confined spaces like tombs.

Advancements in noninvasive mobile analytical instruments with complementary capabilities have significantly enhanced in situ examination of cultural heritage.^1,2^ Typically, the identification of heritage materials at a close range employs a suite of complementary analytical techniques including VIS-NIR (400–900 nm) reflectance spectroscopy, Raman spectroscopy, and X-ray fluorescence (XRF) spectroscopy. VIS-NIR reflectance spectroscopy interrogates electronic transitions of molecules, while Raman spectroscopy probes vibrational transitions of molecules, providing highly specific identification of the molecular structure, and XRF assesses the elemental composition. Laser-induced breakdown spectroscopy (LIBS), a microdestructive technique, also provides in situ elemental analysis through minimal material ablation, creating a plasma that emits spectral lines indicative of atomic transitions. LIBS has the advantage of being able to probe all elements while XRF struggles to detect light elements (*Z* < 13) in air even when operated in contact with a material.

The earliest reported combined use of LIBS and Raman spectroscopy was in artworks analysis, leveraging the complementarity between the two techniques for pigment identification.^3^ Since then, the complementary use of the laser-based spectroscopies is increasingly recognized, not only in cultural heritage^4−7^ but also in mineralogy, pharmaceutical, and marine studies.^8−10^ While the microdestructive nature of LIBS could limit its use on cultural heritage assets, its ability to perform depth-resolved elemental analysis through successive laser pulses at the same sample location proves invaluable. Traditional investigation of wall painting stratigraphy relies on time-consuming sampling and preparation of cross sections followed by the microscopic examination, whereas LIBS allows for immediate, in situ, and controlled analysis through adjusting laser parameters.^11^ At close range (centimeters), mobile systems combining Raman spectroscopy and LIBS have been employed for depth profiling in ancient bronzes^12^ and paintings.^13,14^

Existing methodologies primarily operate at close-ranges (<30 cm), where scaffolding is required for access, which often lacks the stability necessary for sensitive measurements.^15−17^ For instance, Raman measurements typically require long integration times, necessitating vibrational stability over seconds to minutes, a requirement that depth profiling with LIBS also shares to ensure that consecutive ablations can be conducted at the same spot.

To address these limitations, remote sensing instruments that can be deployed easily on stable ground have been developed in our group, enabling analysis of different areas at distances ranging from a few to tens of meters without relocating instruments or operators. Our research has led to the creation of the remote standoff reflectance spectral imaging system, PRISMS^18,19^ and a remote standoff Raman spectroscopy system customized for cultural heritage research.^20,21^ These systems have been applied successfully across various heritage sites, primarily for wall painting analysis.^20,22,23^ Remote standoff LIBS have been developed by other groups for heritage applications to investigate stones, metals, ceramics, and effects of remote laser cleaning.^24−27^ We have recently also developed a remote standoff LIBS system to provide complementary elemental information.^23^

The complementary application of remote standoff LIBS and Raman spectroscopy has shown promise in facilitating material identification in geological and planetary sciences^28,29^ and for explosives detection.^30^ However, no prior work has been reported on using LIBS-based multimodal remote sensing for stratigraphic assessment at significant standoff distances (>1 m), apart from our preliminary laboratory test of a mock-up layered paint sample.^23^

This paper presents the first multimodal remote sensing system that integrates LIBS, Raman spectroscopy, and reflectance spectral imaging, enabling in situ stratigraphic analysis of wall paintings at standoff distances on the order of 10 m. The feasibility and effectiveness of the system are demonstrated through laboratory tests and in situ stratigraphic analysis of whitewashed historical wall paintings. This system offers a valuable tool for heritage science, with broader applications in other scientific and industrial domains requiring the characterization of stratigraphic chemical composition from a distance.

## Experimental Section

### Multimodal Remote Standoff Spectroscopy System

Building on our prior development of remote standoff reflectance spectral imaging (PRISMS)^18,19^ and Raman spectroscopy systems for noninvasive material analysis of wall paintings,^20,21^ the addition of remote LIBS enriches our suite of remote spectroscopy techniques.

**Figure 1:**
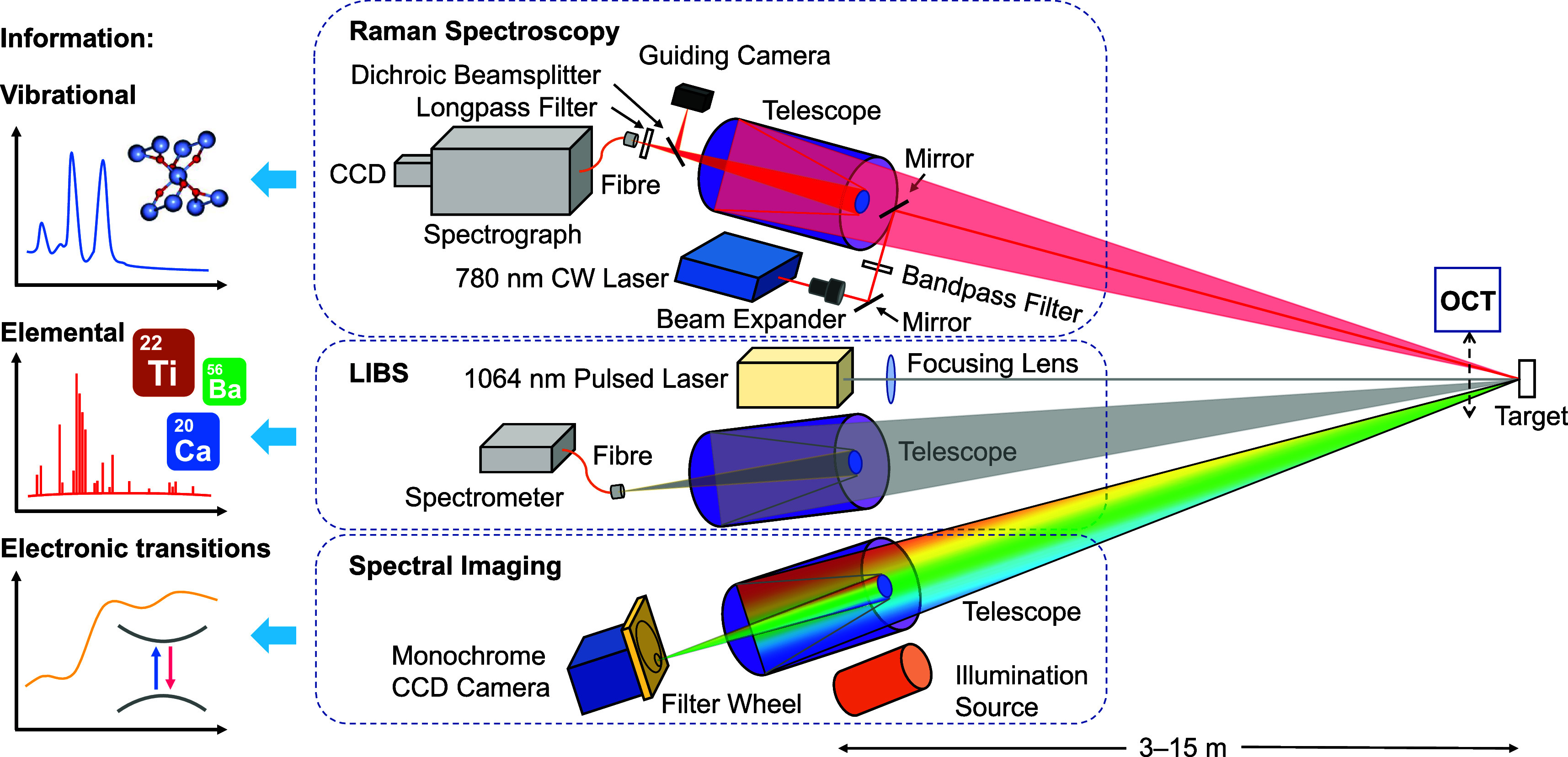
Schematics of the multimodal remote standoff spectroscopy and imaging system consisting of LIBS, Raman spectroscopy, and reflectance spectral imaging using white light illumination. Optical coherence tomography (OCT) was used in laboratory tests to monitor ablation crater formation during LIBS analysis.

The remote multimodal depth-resolved analysis setup includes three instruments operational at standoff distances of order 10 m: remote standoff LIBS, Raman spectroscopy, and VIS-NIR reflectance spectral imaging (Figure 1). The design prioritizes specific requirements for in situ analysis of heritage objects, such as minimizing laser-induced material alteration, ensuring instrument mobility, and enabling practical data collection under field conditions. While a single pulsed laser could be used for a coaligned Raman and LIBS system where high energy enables ablation and low energy supports Raman analysis, our prior studies^20,21^ have shown that low-energy pulsed lasers at safe limits from laser-induced degradation are highly inefficient in producing Raman spectra with adequate signal-to-noise ratios. Continuous wave (CW) lasers are therefore preferred for Raman spectroscopy due to their superior performance under these conditions.

Both Raman and LIBS require high spectral resolution, but their distinct operational requirements make sharing a spectrometer impractical. Raman spectra typically cover a wavelength range of ∼100–300 nm, starting from the Rayleigh line, while LIBS signals span a fixed yet broader range of ∼200–850 nm. LIBS probes atomic transitions on microsecond time scales, necessitating a fast detector, whereas Raman spectroscopy, being ∼6 orders of magnitude less efficient than reflectance spectroscopy, requires integration time of seconds to minutes and hence a cooled and slow detector to minimize dark noise and readout noise. The need to optimize spectral sensitivity for different wavelength regions further supports the decision to use separate spectrometers equipped with dedicated gratings and detectors for each technique. Therefore, the final system configuration includes distinct remote standoff Raman, LIBS, and VIS-NIR reflectance spectral imaging systems, arranged with the LIBS system at normal incidence to the sample, while Raman and spectral imaging systems positioned next to it at angles of <4° (Figure 1).

A 780 nm CW laser (Newport TLB-7113-01) for Raman spectroscopy and a 1064 nm pulsed laser (Continuum Minilite ML II) for LIBS were focused onto the sample, achieving an irradiance of 5.5 W/cm^2^ for Raman spectroscopy with a focused spot of approximately 1 mm, and a peak intensity of 0.56 GW/cm^2^ for LIBS with a spot size of around 1.5 mm. This configuration was designed to ensure accurate material identification in the ablation crater, while minimizing laser-induced damage by maintaining LIBS intensity just above the ablation threshold. Raman spectra were collected using a Maksutov–Cassegrain telescope (Meade ETX 90), filtered to eliminate the Rayleigh line, and directed to a Czerny-Turner spectrograph (Andor Shamrock 193i) coupled with a cooled CCD detector (Andor iDus 416), achieving a spectral resolution of ∼4 cm^–1^ for 140–1300 cm^–1^ or ∼8.5 cm^–1^ for 140–3300 cm^–1^. For LIBS, a separate telescope (Meade ETX 90) collected the emitted light, transmitting it via an optical fiber to a four-channel spectrometer system (Avantes AvaSpec-ULS2048CL-EVO), covering 200–945 nm with resolutions from 0.10 to 0.28 nm. In situ analysis employed a compact spectrometer (Avantes AvaSpec-ULS2048CL) configured for 364–925 nm with a spectral resolution of ∼0.5 nm. The remote standoff spectral imaging system operational over 3–30 m consists of 10 spectral bands of bandwidth 40 nm with central wavelengths at 50 nm intervals from 400 to 850 nm.^18,19^ A Tungsten source projected remotely provides broadband illumination.

The daylight subtraction procedure developed in our earlier work^20^ for standoff Raman spectroscopy is normally followed where necessary. However, all of the work presented here happened to be conducted in darkness. Further details of instrument configuration, experimental parameters, and data processing can be found in the Supporting Methods section in the Supporting Information.

## Results and Discussion

### Laboratory-Based Evaluation of the System using a Mock-Up Sample

**Figure 2:**
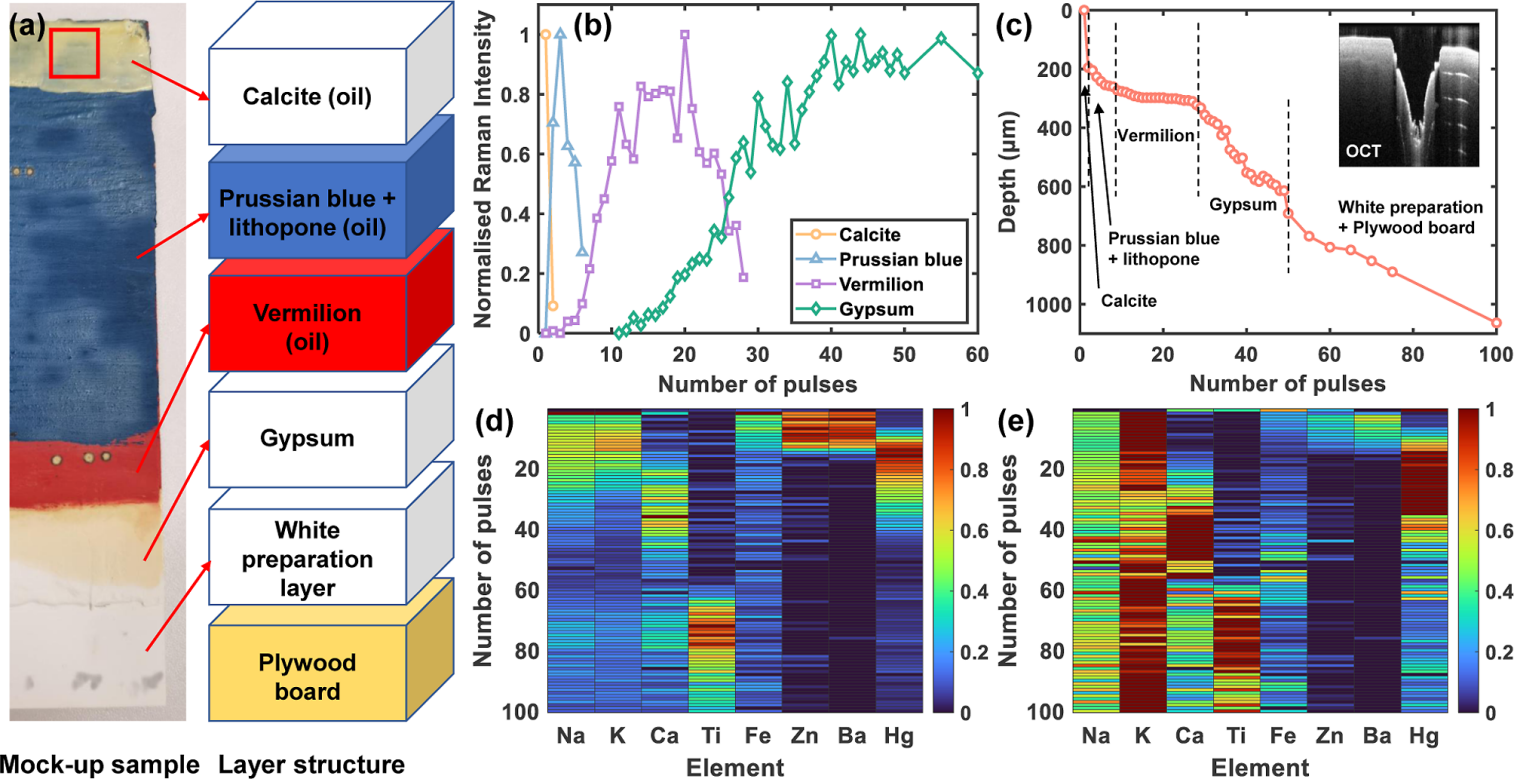
Depth-resolved characterization of the multilayer mock-up paint: (a) paint stratigraphy; (b) evolution of molecular composition of paint layers represented by each pigment’s characteristic Raman peak (calcite: 1086 cm^–1^, Prussian blue: 2155 cm^–1^, vermilion: 253 cm^–1^, and gypsum: 1008 cm^–1^) during the first 60 LIBS laser pulses; (c) ablation depth extracted from OCT images, inset shows the ablation crater formed after 100 laser pulses; (d) depth-normalized LIBS elemental distribution; and (e) spectrum-normalized LIBS elemental distribution. The normalized LIBS heatmaps are color coded from 0 to 1 in increasing intensity.

Prior to depth-resolved material identification of wall paintings at heritage sites, the performance of the remote standoff multimodal spectroscopy system was evaluated under laboratory conditions, using a multilayer mock-up paint sample with typical pigment combinations found on 19th century English wall paintings, mounted at ∼6 m from the instruments. The sample, prepared on a plywood board coated with a white preparation layer identified to be rutile (TiO_2_) and chalk (CaCO_3_) with Raman spectroscopy, consisted of paint layers from bottom to top of gypsum (CaSO_4_·2H_2_O), vermilion (HgS), a mixture of Prussian blue (KFe[Fe(CN)_6_]·H_2_O) and lithopone (coprecipitate of BaSO_4_ and ZnS), and calcite (CaCO_3_) (stratigraphy is shown in Figure 2a). All layers were painted 1 year prior to this study, with linseed oil serving as the binding medium except for the gypsum layer.

Optical coherence tomography (OCT), a technique for noncontact and noninvasive imaging of subsurface microstructure of translucent material, was employed at close range (1 cm) to examine the morphological evolution of the ablation crater by laser pulses.^31,32^ OCT is not required for the stratigraphic analysis; it is only used in the laboratory experiments to estimate the ablation rates and the visualization of the ablation process. For multimodal analysis, sequential interrogation by reflectance spectral imaging, Raman spectroscopy, and OCT and LIBS was applied in each experimental cycle, with noninvasive techniques preceding the microdestructive LIBS. Reconstructed color images from PRISMS visually captured the ablation process, revealing the colors of the consecutively ablated layers inside the ablation crater. Reflectance spectra extracted from the same spot were compared to the reference data of standard materials. The Kubelka–Munk (KM) modeling was employed to assess possible mixtures of painting materials.^1,33^

Depth-resolved composition was monitored through the signal evolution of each technique (Movie S1) during the pulse-by-pulse laser ablation process. A total of 100 pulses were delivered. After pulse #040, Raman spectra stopped evolving, which informed the cessation of Raman measurements after pulse #060 until the end of the final pulse (#100). For Raman spectroscopy, calculations of net peak counts above the fluorescence background were performed to determine the intensity evolution of characteristic Raman signals of each material throughout the ablation process (Figure 2b). Physical depths of the ablation crater were extracted from OCT images following each laser pulse (Figure 2c). The depths of the crater formed as a function of the number of pulses demonstrated variations in the ablation rates as different painting materials were reached. The net counts of LIBS emission lines over the continuum background were extracted. Heatmaps extracted from normalized intensities of representative emission line per element were introduced to visualize the transitions of paint layers (Figure 2d,e). Normalization is performed along two dimensions for LIBS data cubes, offering: (1) depth normalization: intensities of each emission line versus pulse number are normalized to be read element by element, which reveals the elemental distributions in depth; (2) spectrum normalization: intensities of the representative emission lines of all the elements in a single spectrum are normalized, which indicate the dominant elements in each pulse and preserve the ratios of line intensities. The depth normalization is used to profile the evolution of intensities of individual elements, while the spectrum normalization is used to determine the relative elemental composition in each spectrum. The relative intensities of different elements should not be directly compared in depth normalization. Similarly, the relative intensities of one element in different shots should not be compared in spectral normalization. By combining information from both normalizations, the layer-by-layer elemental composition can be better elucidated.

Overall, the evolution patterns of Raman spectroscopy and LIBS were largely consistent, although a temporal offset was observed. Raman signals typically detect new layers earlier than LIBS, likely resulting from the deeper penetration depth of the 780 nm Raman laser compared to the thinly ablated layer in LIBS. For instance, the ablation rate in the vermilion layer, calculated based on the results of the OCT analysis, was ∼1 μm/shot, while the 780 nm Raman spectroscopy is much greater than 1 μm. When LIBS signals were emerging from the surface, Raman spectroscopy might start to reveal information from deeper layers. Furthermore, subsequent pulses continued to ablate residual materials from the wall of the ablation crater (Figure 2c), showing elemental information from preceding layers.

**Figure 3:**
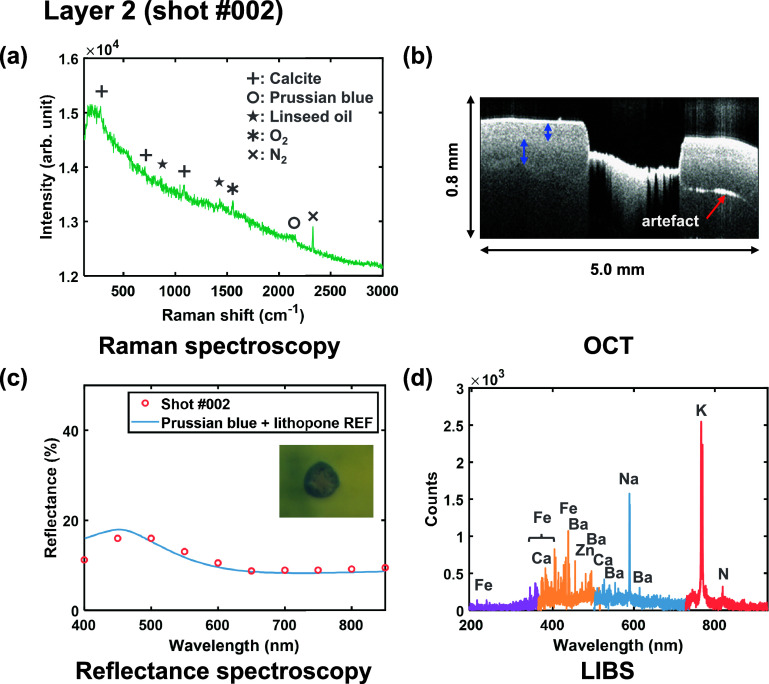
Material analysis of layer 2 (shot #002). (a) Raman spectrum before the second shot. (b) OCT virtual cross-section image of the ablation crater, with red and blue arrows marking an instrumental artifact and the top two paint layers, respectively. (c) Reflectance spectrum, obtained by spectral imaging, from the blue area (inset), fitted with a Kubelka–Munk model using the mixture of standard Prussian blue and lithopone reflectance spectra. (d) LIBS spectrum from the second shot. Raman, OCT, and spectral imaging were performed before the LIBS shot #002.

In the first cycle (Figure S1), Raman detected strong calcite signals (1086 and 280 cm^–1^), alongside linseed oil peaks (1442 and 1304 cm^–1^) (Figure S1a).^34,35^ OCT images (Figure 3b) revealed that the first shot ablated a significant amount of materials (∼200 μm in depth). However, LIBS detected no strong emission lines (Figure S1d), suggesting that the primary effect of the first shot was dominated by fragmentation and sublimation, rather than thermal vaporization, preventing sufficient plasma generation. Aided by the true color image extracted from spectral imaging (Figure 3c), it appeared that the calcite layer had been fully penetrated, exposing the Prussian blue/lithopone layer. The Raman signal at ∼2155 cm^–1^ collected before the second shot (Figure 3a) confirmed the presence of Prussian blue. Weak signals corresponding to calcite and linseed oil were still present, likely from residual materials at the edge of the ablation crater. No lithopone signal was detected by Raman spectroscopy, probably because of the low concentration of it in the mixture. However, LIBS detected K, Fe, Ba, and Zn, the main elements of Prussian blue and lithopone, at the same shot (Figure 3d). The reflectance spectrum extracted from the blue ring at the bottom of the ablation crater was in good agreement with the Kubelka–Munk model fit using a mixture of reference Prussian blue and lithopone spectra, corroborating the LIBS and Raman results. For the subsequent layers, the elemental and various molecular information continued to demonstrate complementary use, revealing characteristic properties of vermilion and gypsum (Figures S2 and S3). Detailed results of the multimodal analysis can be found in Movie S1.

Overall, the system successfully distinguished all paint layers present in the mock-up sample. The identification of lithopone within the blue layer of the Prussian blue and lithopone mixture was challenging, likely due to its low concentration in the mixture.

### In Situ Analysis of Whitewashed Wall Paintings

The laboratory evaluation demonstrated the effectiveness of the multimodal remote standoff spectroscopy system for depth-resolved material characterization of a sample with a complex stratigraphy, paving the way for its application at heritage sites. A field campaign was conducted for in situ analysis of historical wall paintings in the Unity Chapel of the Cathedral Church of St Barnabas in Nottingham, UK, where the original 19th century decorative scheme by Augustus Pugin (1812–1852), a leading figure in Gothic Revival architecture, had been covered by whitewashing owing to changes in aesthetic taste. The proof-of-concept field campaign showed how the remote multimodal spectroscopy approach can be used to reveal the original mural stratigraphy and subsequent paint schemes.

**Figure 4:**
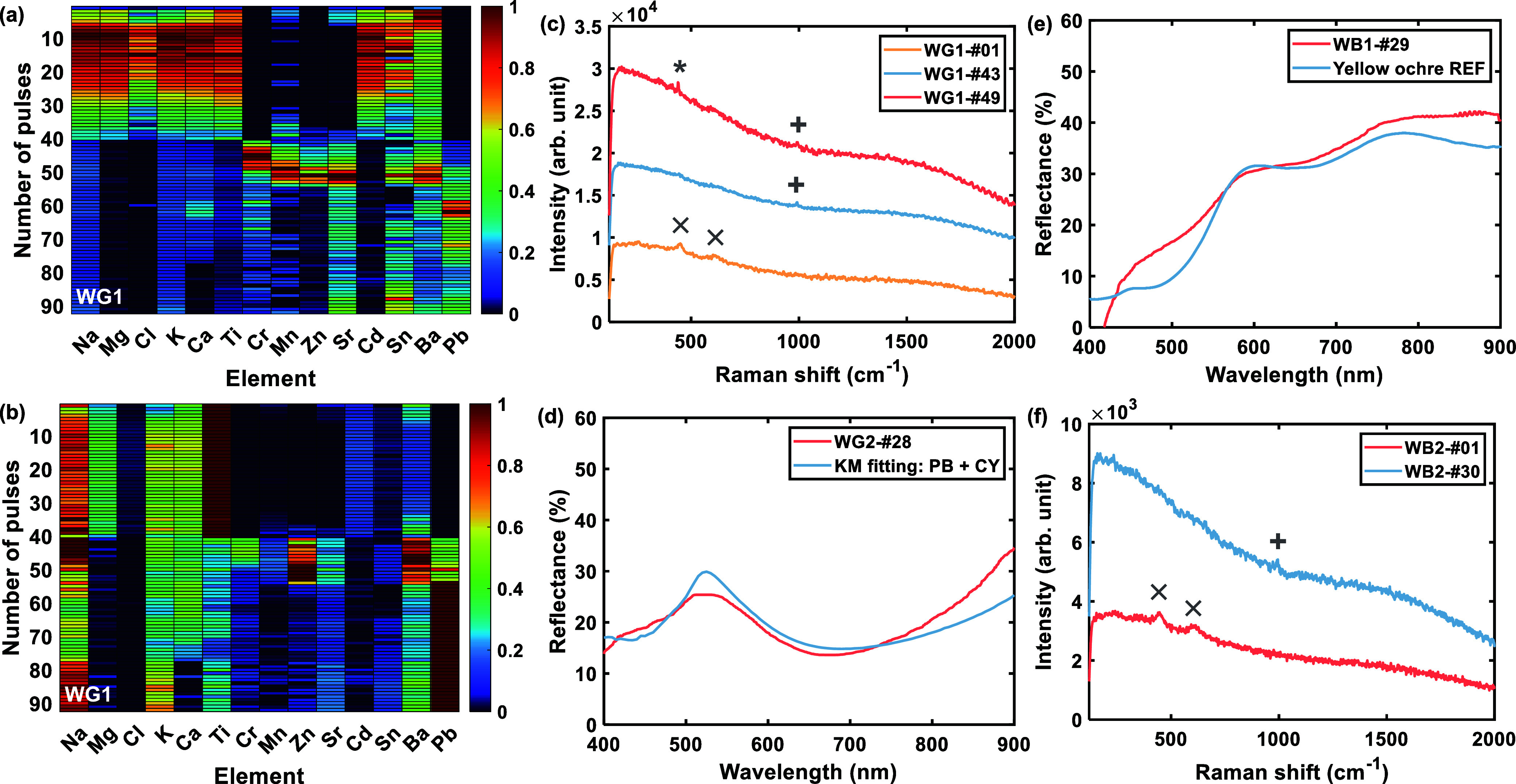
Spectroscopic information during ablation: (a) Depth-normalized LIBS evolution of WG1; (b) spectrum-normalized LIBS evolution of WG1; (c) Raman spectra from WG1; (d) reflectance spectrum of a green layer in WG2 fitted with KM modeling using a mixture of Prussian blue (PB) and chrome yellow (CY); (e) reflectance spectrum of a brown layer in WB1, compared with the reference spectrum of yellow ochre; and (f) Raman spectra from WB2. The normalized LIBS heatmaps are color coded from 0 to 1 in increasing intensity. Raman signals: rutile (×), BaSO_4_ (+), and ZnO (*).

**Figure 5:**
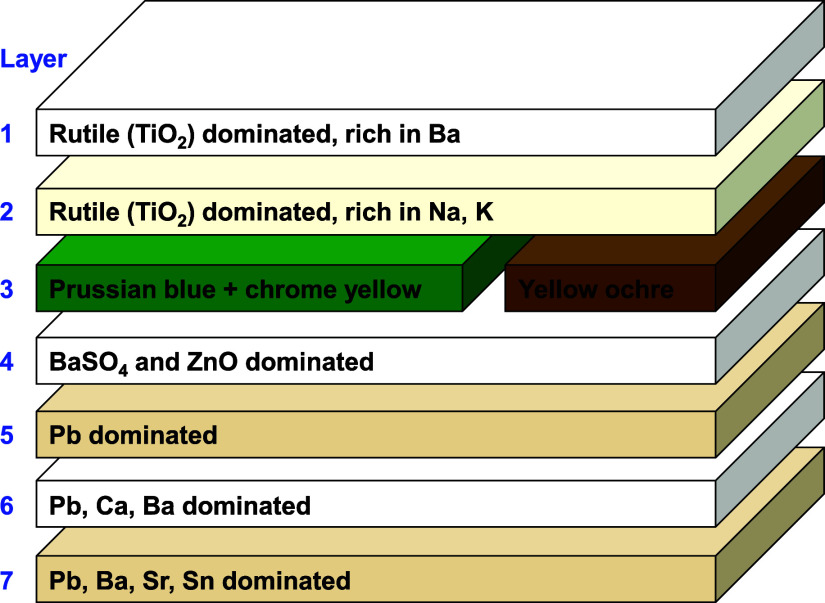
Layer interpretation of the wall painting structure in Unity Chapel based on multimodal analysis results.

The remote standoff multimodal spectroscopy system was set up (Figure 1) for in situ analysis of four positions (WG1, WG2, WB1, and WB2) across the whitewashed southeast wall of the Unity Chapel at ∼7 m distance. Given the time limitation of the on-site campaign, Raman and reflectance spectroscopies were performed only when significant changes in LIBS spectra were observed. The depth-resolved elemental composition obtained from LIBS evolutions (Figures 4a,b and S4) indicated largely consistent layer structures at different sample spots. Multimodal spectroscopic data from different data sets (Figure 4) revealed similar composition of the layers, except for the polychrome layer beneath the whitewash where either green or brown paint was found, suggesting both paints belong to the same layer, most likely the surface of the original scheme covered by the whitewash. In total, at least seven distinct layers were identified based on the results of the complementary analysis. The stratigraphy is summarized in Figure 5.

Raman analysis identified rutile (TiO_2_) before the first LIBS pulse, with signals at 450 and 609 cm^–1^ (Figure 4c,f). Correspondingly, LIBS spectra from the first few shots revealed intense Ti emission lines alongside Ca, Ba, Na, K, Mn, Sr, Sn, Cl, Cd, and Mg signals. The depth evolution patterns for these elements at the four positions were largely consistent. It is evident in almost all data sets that Ba signals peaked earlier than the others, implying more than one layer of the whitewash. The presence of Ba hints at BaSO_4_, commonly used as an extender in white paints.^36^ Layer 1 is therefore defined as a Ba-rich rutile layer at the top part of the whitewash.

Layer 2 features a considerable amount of alkali (Na and K) and required more pulses to ablate compared to layer 1. Traces of Cd in this layer might be attributed to the addition of a small amount of cadmium yellow or red, imparting a slightly warmer tone to the whitewash.

Layer 3 exhibits two different paint colors as the laser pulses fully penetrated the whitewash. In WG1 and WG2, prominent Cr signals emerged immediately after the whitewash layers, which was corroborated by observation of an exposed green layer and a broad band centered at ∼520 nm in corresponding reflectance spectra (Figure 4d). This spectral shape was not found in any known Cr-containing green pigment. KM modeling using various potential mixtures of pigments consistent with the elemental results given by LIBS showed that a mixture of Prussian blue (KFe[Fe(CN)_6_]·H_2_O) and chrome yellow (PbCrO_4_) was the best fit. Pb was present in this green layer and was detected at the emergence of Cr signals (as demonstrated in the spectrum-normalized evolution plots in Figures 4b and S4b), supporting chrome yellow presence in this paint layer. It was a common practice in the 19th century to mix Prussian blue with chrome yellow to achieve a green hue.^36^ In WB1 and WB2, brown paint emerged after the ablation of the whitewash layers. Spectral reflectance demonstrated characteristic spectral features of yellow ochre [FeO(OH)] (Figure 4e).

Layer 4, underlying both green and brown paints, contains Ba, Zn, Na, Mn, Sr, Sn, Ca, and K (Figures 4a and S4a). Raman signals at 990 and 439 cm^–1^ confirmed the presence of BaSO_4_ and ZnO, a common white mixture,^37^ suggesting a white substrate layer beneath the colored paint layer (Figure 4c).

Layer 5 is characterized by dominant Pb signals in spectrum-normalized LIBS evolutions (Figures 4b and S4b,d). Modest levels of Ca, Na, and K were also detected. Owing to time constraints, Raman measurements were selectively conducted only when a distinct change was observed during the investigation. However, no Raman data were obtained for deeper layers where no clear color transition was noted.

Layer 6 exhibits intense Pb and Ca signals along with Ba, Na, K, and Sr. Pb signals in this layer are significantly stronger than that in the preceding layer, indicating a different material composition.

The deepest layer reached (layer 7) in this investigation is characterized by increasing Na, K, Sr, and Sn signals. The maximum number of pulses delivered at all examined sites was 90 (in WG1), at which point the LIBS spectra ceased evolving, and the investigation was terminated.

Overall, the results suggest a stratigraphy of at least seven layers, in which a polychrome paint scheme is present beneath at least two whitewash layers in the investigated area. The green paint is most likely a mixture of Prussian blue and chrome yellow, while the brown paint is most likely a yellow ochre.

### Verification of Mural Stratigraphy

**Figure 6:**
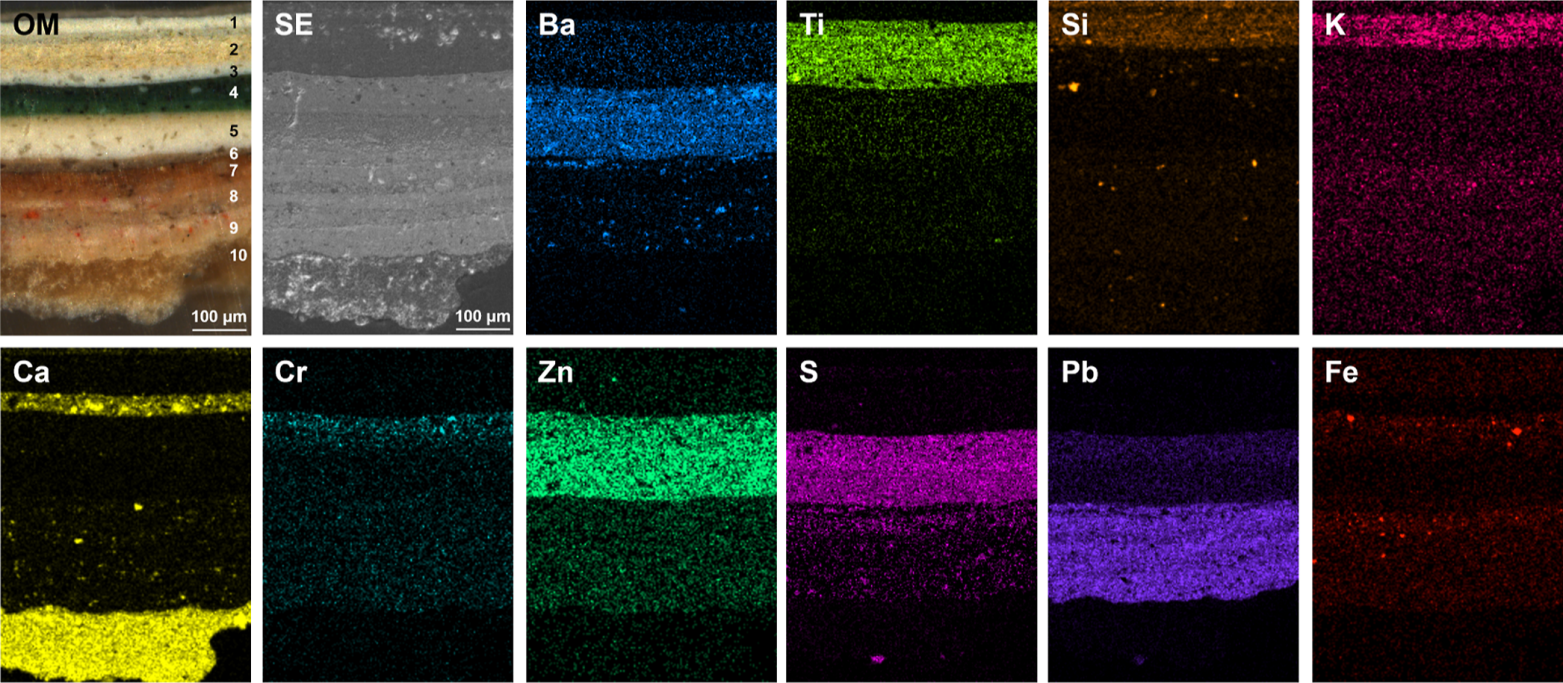
OM image, SEM secondary electron (SE) image, and EDS elemental maps of the cross-section sample obtained near WG sites.

Samples from the same wall painting area were extracted during a conservation campaign, offering an invaluable independent verification of our multimodal stratigraphic investigation. Two cross-sectional samples obtained from areas adjacent to the ablation craters in green and brown polychrome regions were embedded in resin, polished, and subjected to a stratigraphic analysis using optical microscopy (OM) and scanning electron microscopy (SEM). Both samples exhibited consistent stratigraphy (Figures 6 and S5). The elemental distribution revealed by energy-dispersive X-ray spectroscopy (EDS) largely concurred with our LIBS results.

Based on OM and EDS analysis, the whitewash consists of at least three layers. From top to bottom, the first layer contained less Ti compared with deeper layers, while Ba exhibited a rather homogeneous distribution throughout the whitewash. This is consistent with our remote multimodal investigation, which indicated a Ba-rich rutile layer in the first stratum. EDS maps showed Si and K in the top two layers, while Ca was prominent in the third layer but weak in the first. A closer inspection of the WG1 data set revealed that in the depth-normalized LIBS evolution, K, Na, and Mg peaked at the 10th shot, while Ca peaked at the 15th shot (Figure 4a), indicating three whitewashed layers. However, the other three data sets did not delineate stratification as clearly, suggesting that the whitewash stratigraphy in both cross-section samples is more comparable to WG1. The Ca-rich layer in the other three examined sites in our remote investigation might have been too thin to differentiate from the layer above.

The EDS maps revealed the presence of Cr within the green paint layer and Fe in the brown layer. The findings corroborated the identification of Cr in the color layer through LIBS in WG sites and aligned with the characterization of an ochre layer obtained via reflectance spectroscopy at WB sites. Beneath the color paint layer, a thick white substrate rich in Ba, Zn, and S was identified, corroborating the detection of Ba and Zn by LIBS and BaSO_4_ and ZnO by Raman spectroscopy. A modest Pb presence was detected by LIBS in the same layer, as shown in spectrum-normalized evolution plots (notably WG1 and WG2 in Figures 4b and S4b). Deeper layers were mainly rich in Pb and Ca in EDS maps, consistent with LIBS results. A thin reddish layer contained Fe, which LIBS failed to detect. The detection of elements with emission lines in the UV region, including Fe and Si, were difficult due to UV absorption by the aberration correction glass of the telescope in our LIBS setup. The plaster substrate at the base was not reached in our multimodal investigation since the characteristic Ca was not detected by the end of the laser ablation with 90 pulses for WG1.

In total, 9–10 layers were discernible in the cross-section samples via OM and SEM images and EDS maps. Our remote standoff multimodal approach identified 7–8 layers. The main discrepancy arose from the insufficient number of pulses delivered in our remote investigation to reach the plaster substrate. Elements such as Cl, Mn, Cd, Sn, and Sr were successfully identified in LIBS spectra but not by EDS. Na and Mg were not detected by EDS due to the decision to perform the EDS at a 20 kV voltage to optimize overall sensitivity for elements present in these samples.

## Conclusions

This study presents a novel mobile multimodal remote stand-off spectroscopy system combining LIBS, Raman spectroscopy, and reflectance spectral imaging for in situ wall painting research. For the first time, depth-resolved identification of the chemical composition of wall painting stratigraphy at a distance of order 10 m was achieved by synergistically leveraging elemental, molecular vibrational, and electronic transition signatures. The instruments were deployed and operated from stable ground without the need of scaffold platforms.

Following laboratory evaluation, a field campaign at the Unity Chapel of the Cathedral Church of St Barnabas in Nottingham, UK, successfully revealed at least seven distinct paint layers in the whitewashed historical wall painting, demonstrating the effectiveness of this remote standoff multimodal approach. The depth-resolved composition of the mural stratigraphy was further validated by SEM-EDS microanalysis of physical samples from the same wall.

This methodological advance provides a valuable tool for wall painting research and broader applications in in situ analysis of layered cultural heritage materials. The approach can be applied to general architectural survey, sculptures, and monitoring of salt damage to buildings. Additionally, it holds significant potential for applications in a variety of industrial and scientific domains, including industrial asset monitoring and other remote sensing areas where in situ stratigraphic characterization of complex systems is required.

## Supporting Information Available

The following files are available free of charge. The Supporting Information is available free of charge at https://pubs.acs.org/doi/10.1021/acs.analchem.4c05264.

Additional experimental methods, multimodal material analysis of paint layers in the mock-up sample, heatmaps for LIBS evolution, OM and SEM images, and EDS elemental maps of the cross-section sample obtained near WB positions (PDF)
Evolution of color images derived from spectral image cubes captured by the remote standoff reflectance spectral imaging system, PRISMS; OCT images; Raman spectra collected from the remote standoff Raman spectroscopy system; and LIBS spectra collected from the remote standoff LIBS system during the formation of the ablation crater in the laboratory-based evaluation using a mock-up sample (AVI)

## Supplementary Material

ac4c05264_si_001.pdf

ac4c05264_si_002.avi
