# Supplementary material for: Ground-Based Remote Standoff Laser Spectroscopies and Reflectance Spectral Imaging for Multimodal Analysis of Wall Painting Stratigraphy
Source: Anal Chem. 2024 Nov 13;96(47):18907–15. doi: 10.1021/acs.analchem.4c05264 (PMC11603405; doi:10.1021/acs.analchem.4c05264)
Supplement: Supplementary file 1 — ac4c05264_si_001.pdf [file ac4c05264_si_001.pdf]

Supporting Information

Ground-based remote standoff laser spectroscopies and spectral imaging for multimodal analysis of wall painting stratigraphy

Yu Li,[†,‡] Amelia Suzuki,[†] C. S. Cheung,[†] Sotiria Kogou,[†] and Haida Liang*[,†]

† Imaging and Sensing for Archaeology, Art History and Conservation (ISAAC) Lab, School of Science and Technology, Nottingham Trent University, Nottingham NG11 8NS, United Kingdom

‡ Current address: Institute of High Energy Physics, Chinese Academy of Sciences, Beijing 100049, P.R. China

E-mail: haida.liang@ntu.ac.uk

Phone: +44 (0)115 848 8056

Table of Content

Supporting Methods

Supporting Figures

Figure S1. Multimodal material analysis of layer 1 (shot #001).

Figure S2. Multimodal material analysis of layer 3 (shot #018).

Figure S3. Multimodal material analysis of layer 4 (shot #019).

Figure S4. Heatmaps for LIBS evolution. Heatmaps normalised along depth (a,c,e) and spectral (b,d,f) dimensions of LIBS data from the examined sites, WG2, WB1 and WB2.

Figure S5. Optical microscopy (OM) and SEM secondary electron images (SE) and EDS elemental maps of the cross-section sample obtained near WB sites.

Supporting References

Supporting Methods

Laboratory-based evaluation of the system using a mock-up sample

Second order polynomial fittings are performed to 13 known spectral lines from a mercury argon calibration light source (Ocean Optics HG-1) between 790 and 970 nm to establish the wavelength calibration of the spectrometer. Raman spectra were acquired using an exposure time of 40 s and 3 accumulations for automated cosmic-ray removal. Baseline subtraction for Raman spectroscopy is conducted by smoothing the spectrum using a moving median filter, which is efficient in removing broad spectral features such as fluorescence from the original Raman spectrum such that only Raman peaks remain. Alternatively, manual subtraction can be performed using a polynomial fitting in user-defined spectral regions of interest for more complicated situations.

To monitor the formation of the crater by laser pulses, a spectral domain OCT imaging system (Thorlabs CALLISTO) at 930 nm was set up at close range for laboratory evaluation. The system provides an axial resolution of 4.5 μm in paint (assuming a refractive index of 1.5) and a transverse resolution of 9 μm. OCT virtual cross-section images were captured across a 5 mm line segment with a pixel size of 10 × 3.1 μm in width and depth respectively. A motorised linear translation stage was employed to mount the OCT probe to move it back and forth between the crater position and away from the line of sight of the remote/standoff instruments during their operation.

LIBS measurements are conducted in time-integrated mode to capture the entire sequence of emission signals. An in-house written program for automated line assignments has been developed within MATLAB, using LIBS spectral line references from NIST atomic spectral database,[1] complemented by a table of major emission lines of elements relevant to pigment identification.[2] Background subtraction is then

performed to obtain net counts for the emission lines. For the heatmaps demonstrating LIBS evolutions (Figure 2d,e), representative emission lines of identified elements include Ti (453.40 nm), Ca (396.83 nm), Fe (407.14 nm), Ba (455.40 nm), Zn (481.05 nm), Hg (435.83 nm), Na (589.00 nm), and K (766.49 nm). These lines were carefully selected to avoid overlapping with emission signals from other elements.

In situ **analysis of whitewashed wall paintings**

Raman spectra were collected with an exposure time of 200 s and 3 accumulations for automated cosmic-ray removal.

Representative emission lines of identified elements (Figure 4a,b and Figure S5) in LIBS heatmaps include Ti (453.40 nm), Ca (422.69 nm), Ba (553.55 nm), Zn (481.05 nm), Pb (405.78 nm), Na (589.00 nm), K (766.49 nm), Mn (601.99 nm), Sr (460.73 nm), Cr (520.67 nm), Sn (645.36 nm), Cl (837.60 nm), Cd (508.56 nm), and Mg (448.10 nm).

The other experimental parameters were the same as those used in the laboratory testing of the mock-up paint sample.

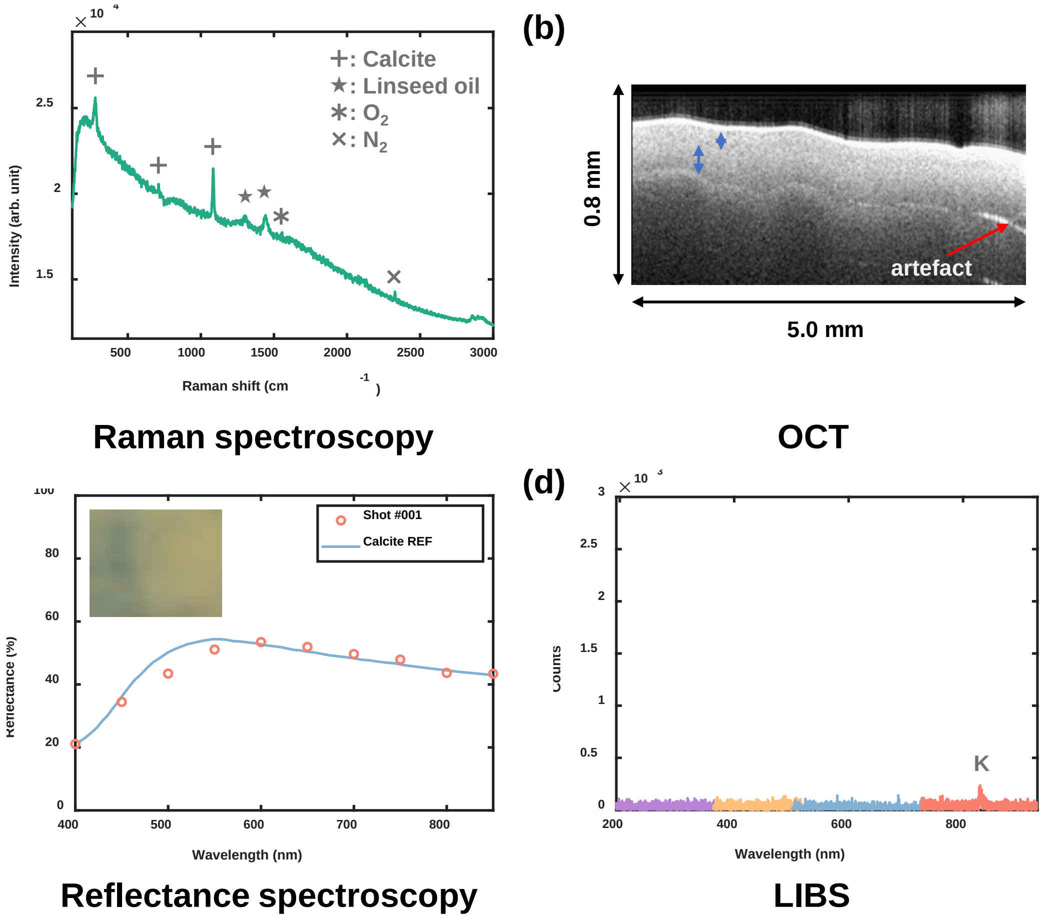

Figure S1. Multimodal material analysis of layer 1 (shot #001). (a) Raman spectrum collected before the 1st shot. (b) OCT image of the region of interest. The red arrow in the OCT image marks an instrumental artefact. The blue arrows mark the two paint layers. (c) The reflectance spectrum, obtained by spectral imaging, extracted from the area as shown in the inset, and plotted with a reference curve of calcite. (d) LIBS spectrum recorded at the 1st shot. The Raman, OCT, and spectral imaging were performed before the LIBS shot #001.

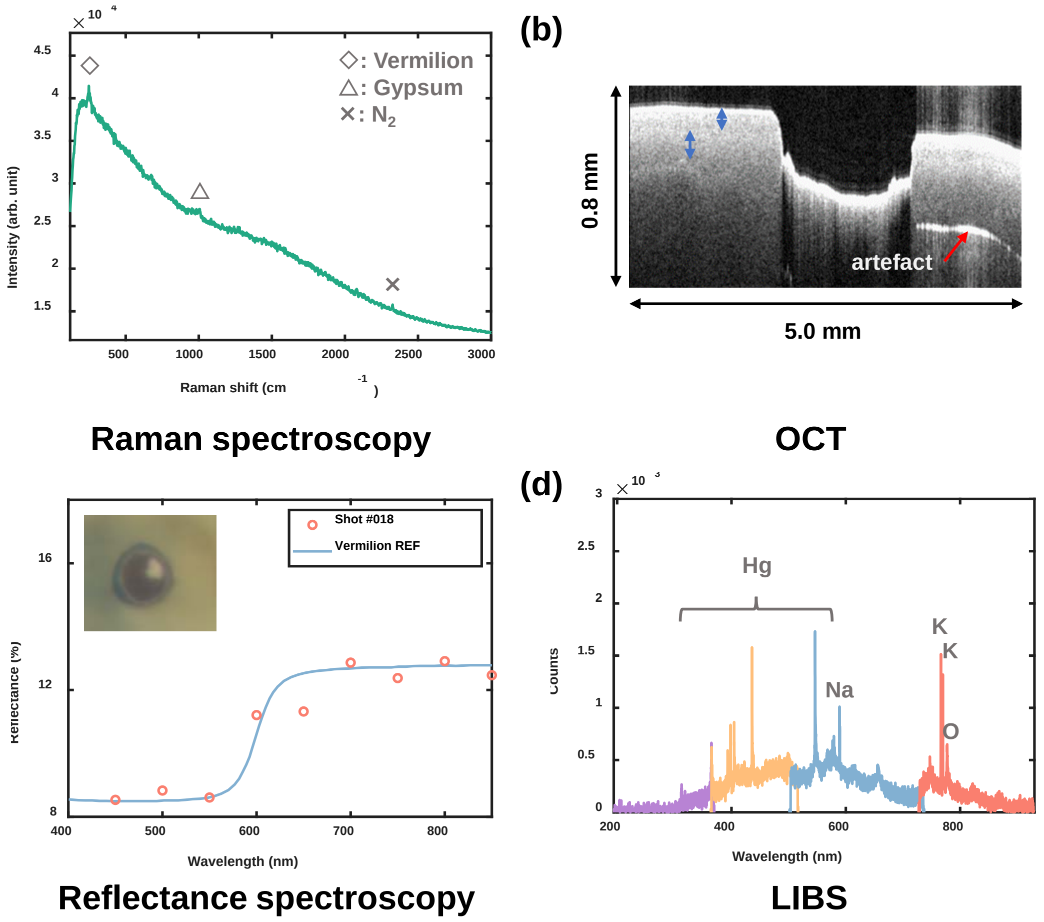

Figure S2. Multimodal material analysis of layer 3 (shot #018). (a) Raman spectrum collected before the 18th shot. (b) OCT image of the ablation crater. The red arrow in the OCT image marks an instrumental artefact. The blue arrows mark the two paint layers. (c) The reflectance spectrum, obtained by spectral imaging, extracted from the red region of the crater as shown in the inset, and plotted with a reference curve of vermilion. (d) LIBS spectrum recorded at the 18th shot. The Raman, OCT, and spectral imaging were performed before the LIBS shot #018.

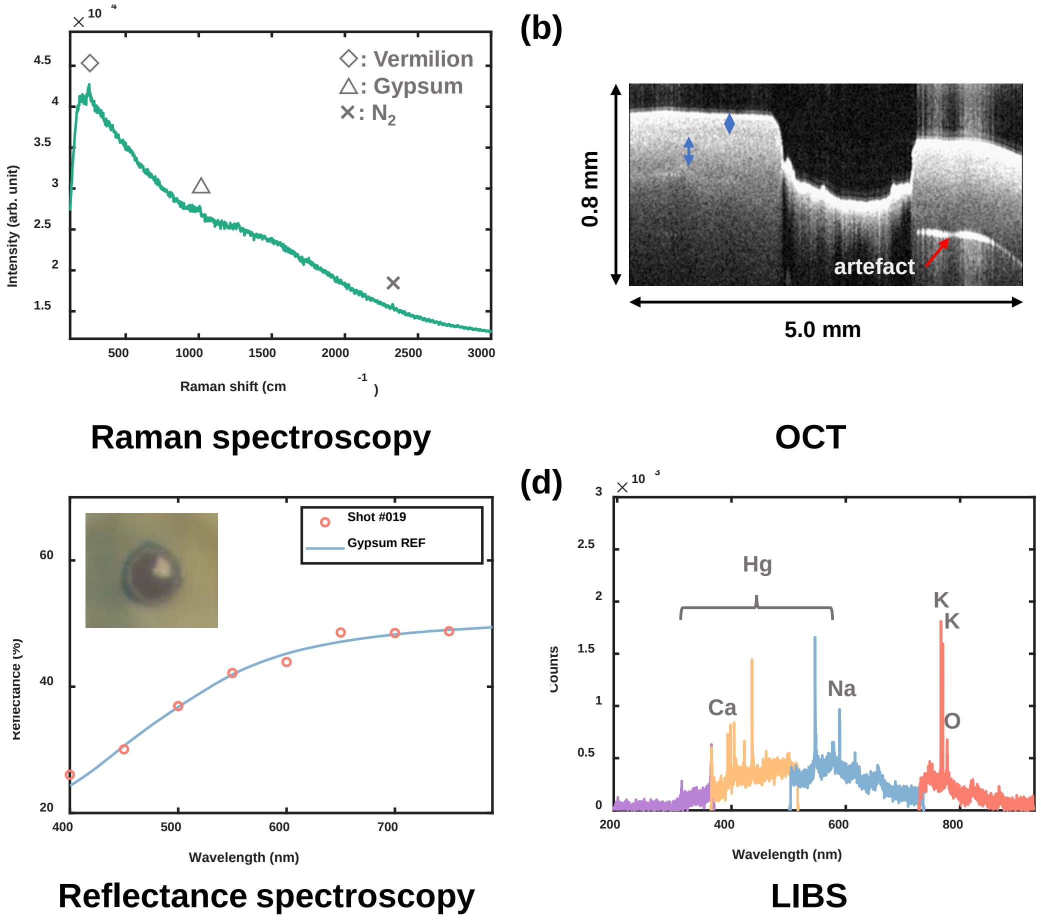

Figure S3. Multimodal material analysis of layer 4 (shot #019). (a) Raman spectrum collected before the 19th shot. Both vermilion and gypsum were detected as the Raman laser spot covered the whole crater. (b) OCT image of the ablation crater. The red arrow in the OCT image marks an instrumental artefact. The blue arrows mark the two paint layers. (c) The reflectance spectrum, obtained by spectral imaging, extracted from the white area at the bottom of the crater as shown in the inset, and plotted with a reference curve of gypsum. (d) LIBS spectrum recorded at the 19th shot. The Raman, OCT, and spectral imaging were performed before the LIBS shot #019.

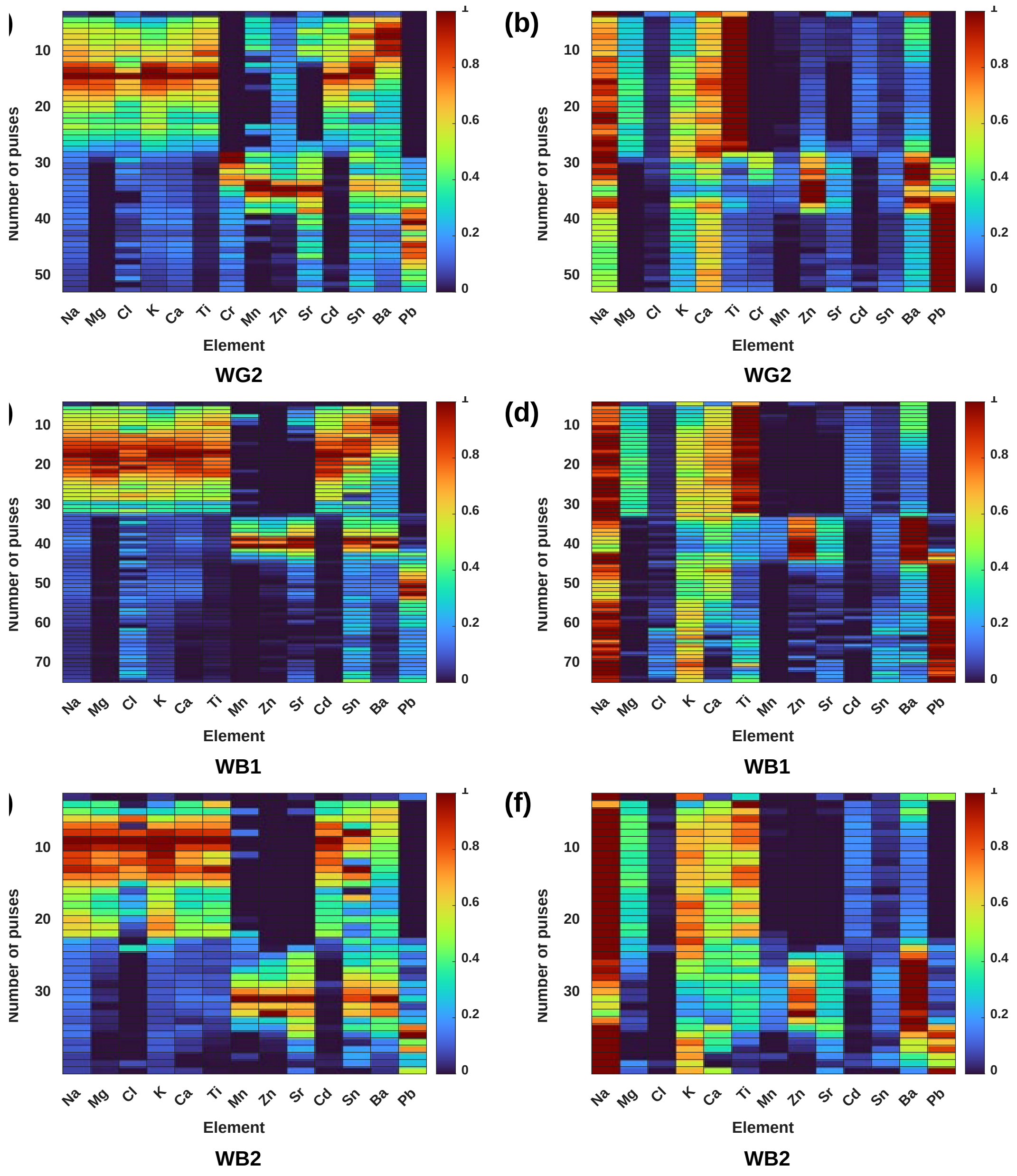

Figure S4. Heatmaps for LIBS evolution. Heatmaps normalised along depth (a,c,e) and spectral (b,d,f) dimensions of LIBS data cubes from the examined sites, WG2, WB1 and WB2. The elements detected are ordered by atomic number. The normalised LIBS heatmaps are colour coded from 0 to 1 in increasing intensity.

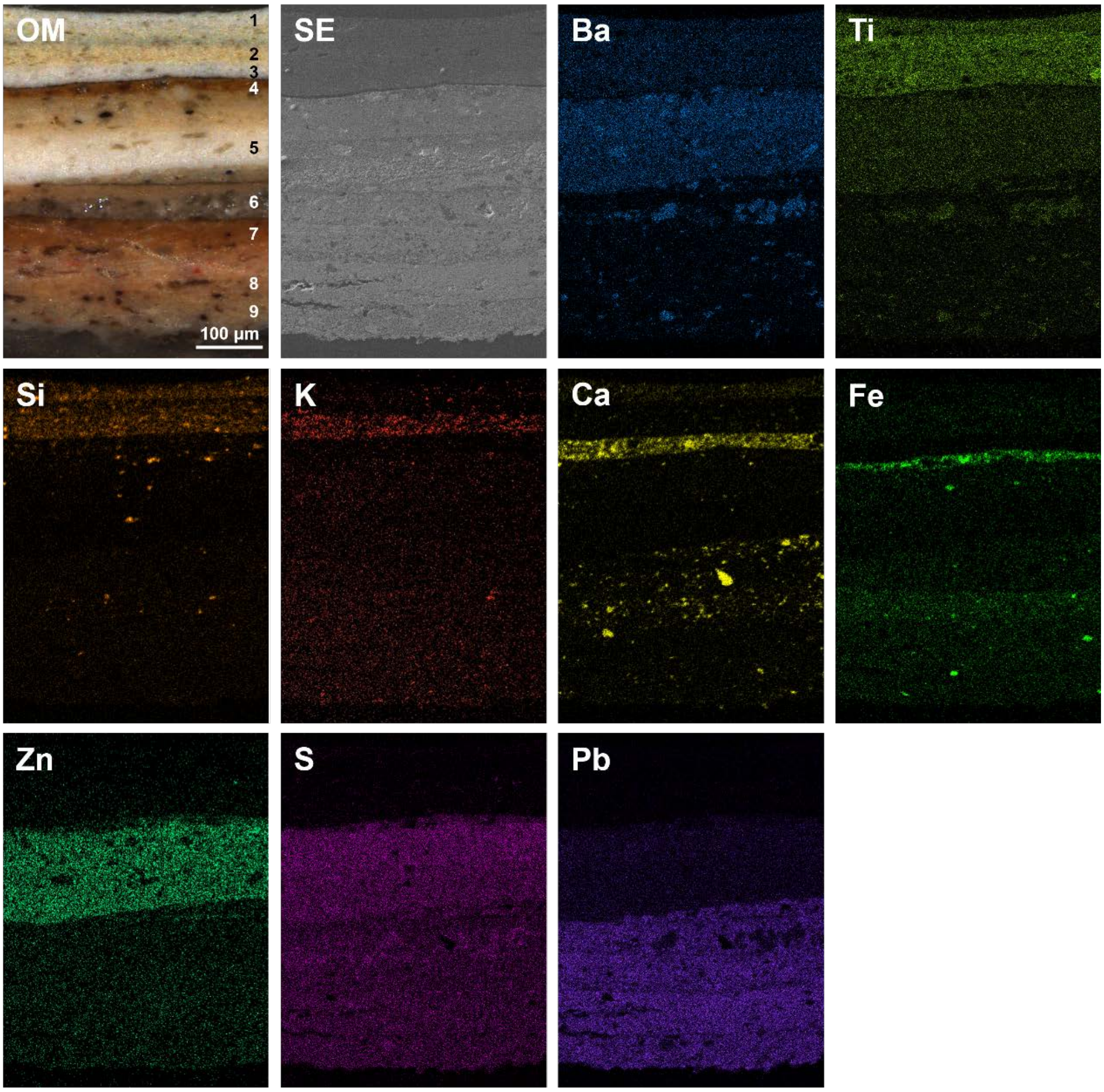

Figure S5. Optical microscopy (OM) and SEM secondary electron images (SE) and EDS elemental maps of the cross-section sample obtained near WB sites.

Supporting References

(1) Kramida, A.; Olsen, K.; Ralchenko, Yu. *NIST LIBS Database*. https://physics.nist.gov/PhysRefData/ASD/LIBS/libs-form.html.

(2) Anglos, D.; Miller, J. C. Cultural Heritage Applications of LIBS. In *Laser Induced Breakdown Spectroscopy*; Cambridge University Press, 2006; pp 332–367.
